# Progress on diagnosis and treatment of multisystem inflammatory syndrome in children

**DOI:** 10.3389/fimmu.2025.1551122

**Published:** 2025-02-19

**Authors:** Zhe Peng, Gang Zhou

**Affiliations:** Department of Pediatric Respiratory Diseases, Chongqing University Three Gorges Hospital, Chongqing, China

**Keywords:** MIS-C, COVID-19, SARS-CoV-2, multisystem inflammatory syndrome, pediatric, Kawasaki disease, immunotherapy, diagnosis

## Abstract

Since the emergence of COVID-19 in December 2019, the novel SARS-CoV-2 virus has primarily affected adults, with children representing a smaller proportion of cases. However, the escalation of the pandemic has led to a notable increase in pediatric cases of Multisystem Inflammatory Syndrome in Children (MIS-C). The pathogenesis of MIS-C is largely attributed to immune-mediated mechanisms, such as cytokine storms and endothelial damage, following SARS-CoV-2 infection. In this review, we comprehensively describe MIS-C, including its definitions as proposed by the CDC, WHO, and RCPCH, which emphasize persistent fever, excessive inflammatory responses, and multi-organ involvement. Additionally, we summarize current treatment approaches, prioritizing immunotherapy with intravenous immunoglobulin and corticosteroids, along with anticoagulation therapy, and monoclonal antibodies in severe cases.

## 1 Introduction

Since the advent of SARS-CoV-2 in December 2019, COVID-19 has escalated swiftly, catalyzing global waves of infection and mortality. Epidemiological investigations indicate a mere 1.7% of these cases involve children (1), attributed primarily to their reduced exposure, less severe symptoms, and lack of comprehensive testing. Typically, pediatric infections manifest with respiratory symptoms and are associated with positive prognoses (2). Nonetheless, a subset of severe pediatric presentations mirroring Kawasaki disease (KD) has surfaced, marked by symptoms such as fever, gastrointestinal distress, cardiac complications, shock, multi-organ dysfunction, and heightened inflammatory markers. Initially identified in the UK during April 2020, analogous cases have subsequently been reported across Europe, the US, and other areas (3–5). Of these severe presentations, 81% demonstrate positivity for SARS-CoV-2 IgG antibodies, whereas 37% show detectable viral nucleic acids (6). Emerging evidence suggests that this syndrome, likely driven by immune responses, follows an encounter with SARS-CoV-2. In response, the World Health Organization (WHO) recognized this condition in May 2020, naming it Multisystem Inflammatory Syndrome in Children (MIS-C) (7). This review delves into the definition, pathogenesis, clinical manifestations, diagnostic criteria, therapeutic options, and prognosis of MIS-C, providing a comprehensive framework for clinicians to manage this syndrome effectively.

## 2 Definition and diagnostic criteria for MIS-C

At present, three distinct definitions of MIS-C have been formulated by the US Centers for Disease Control and Prevention (CDC), the WHO, and the UK’s Royal College of Paediatrics and Child Health (RCPCH). Despite a lack of complete alignment among these definitions, they share several common features: fever, an intense inflammatory response, involvement of multiple organs, either direct exposure to COVID-19 or a prior infection with SARS-CoV-2, and ruling out other infectious etiologies. These criteria are designed to identify children who either fully or partially satisfy the diagnostic requirements for KD (8).

### 2.1 Diagnostic criteria for MIS-c proposed by the RCPCH

(1) Age of onset: All age groups; (2) Clinical features: Persistent fever >38.5°C, accompanied by the dysfunction of one or more organ systems (e.g., gastrointestinal, respiratory, cardiac, renal, neurological) or instances of shock; (3) Laboratory results: Increased levels of C-reactive protein (CRP) and ESR, accompanied by neutrophilia; (4) Epidemiological context: Positive reverse transcription PCR for SARS-CoV-2 (9).

### 2.2 Diagnostic criteria for MIS-C proposed by the WHO

(1) Age of onset: <19 years; (2) Clinical signs: Persistent fever for ≥3 days, plus at least two of the following symptoms: rash or bilateral non-purulent conjunctivitis, mucocutaneous inflammation, hypotension or shock, cardiac dysfunction, diarrhea, vomiting, or abdominal pain; (3) Laboratory indicators: Elevated ESR, CRP, PCT, coagulation abnormalities, and evidence of cardiac injury; (4) Epidemiological context: History of SARS-CoV-2 infection or exposure to COVID-19 (10).

### 2.3 Diagnostic criteria for MIS-C proposed by the CDC

(1) Age of onset: <21 years; (2) Clinical features: Fever >38.0°C for ≥24 hours or subjective fever for ≥24 hours, alongside dysfunction in ≥2 organ systems (gastrointestinal, respiratory, cardiovascular, renal, hematological, dermatological, or neurological); (3) Laboratory findings: Including, but not limited to, one or more of the following: elevated CRP, procalcitonin, ESR, ferritin, interleukin-6, neutrophilia, lymphopenia, and hypoalbuminemia; (4) SARS-CoV-2 infection/exposure within 4 weeks (10). A Boston Children’s Hospital study (11) revealed clinical heterogeneity under the 2020 MIS-C definition, risking misclassification of severe COVID-19 as MIS-C, particularly for cases with respiratory criteria (5, 12, 13). The updated CDC criteria exclude fever duration, renal, neurological, and respiratory involvement (5), improving specificity and aligning more closely with WHO standards (14) (**Table 1**).

**Table 1:** Comparative analysis of MIS-C diagnostic criteria by CDC, WHO, and RCPCH.

Criteria	CDC	WHO	RCPCH
Age	<21 years	<19 years	All age groups
Fever Duration	>38.0°C for ≥24 hours or subjective fever for ≥24 hours	Persistent fever for ≥3 days	Persistent fever >38.5°C
Clinical Features	≥2 organ systems (gastrointestinal, respiratory, cardiovascular, renal, hematological, dermatological, neurological)	At least two of the following: rash or bilateral non-purulent conjunctivitis, mucocutaneous inflammation, hypotension or shock, cardiac dysfunction, diarrhea, vomiting, abdominal pain	Dysfunction of one or more organ systems (gastrointestinal, respiratory, cardiac, renal, neurological) or shock
Laboratory Findings	Elevated CRP, procalcitonin, ESR, ferritin, IL-6, neutrophilia, lymphopenia, hypoalbuminemia	Elevated ESR, CRP, PCT, coagulation abnormalities, evidence of cardiac injury	Increased CRP and ESR, neutrophilia
Epidemiological Context	SARS-CoV-2 infection/exposure within 4 weeks	History of SARS-CoV-2 infection or exposure to COVID-19	Positive reverse transcription PCR for SARS-CoV-2
Strengths	Broad age range allows for inclusive diagnosis; comprehensive laboratory markers enhance specificity	Detailed clinical signs provide clarity; inclusion of coagulation and cardiac injury indicators improves diagnostic accuracy	Simplicity in criteria facilitates quicker clinical decision-making; inclusion of direct PCR testing confirms active infection
Limitations	Overly broad criteria may lead to misclassification; exclusion of certain symptoms (e.g., renal, neurological) may reduce sensitivity	More complex criteria may hinder rapid diagnosis; reliance on history of infection may miss cases with asymptomatic or undocumented exposure	Limited laboratory markers may decrease specificity; reliance on PCR may miss post-infectious cases where viral RNA is not detectable

## 3 Clinical manifestations of MIS-C

The clinical presentation of MIS-C exhibit significant variability and lack specificity, with several overlapping features observed in KD. Common symptoms include persistent fever, mucocutaneous involvement (e.g., swelling of the hands and feet, conjunctivitis, cracked lips, rash), lymphadenitis, and cardiac dysfunction. Multiple organ systems are typically affected, particularly the cardiovascular and gastrointestinal systems, along with the circulatory, respiratory, and neurological systems (15, 16). Recent studies have expanded the symptom spectrum of MIS-C to include acute neurological deficits and severe dermatological reactions (17), renal involvement and hepatic dysfunction (18), and cervical inflammation (19), underscoring its heterogeneous nature.

Based on the predominant clinical manifestations, MIS-C can be categorized into three types: (I) Predominantly persistent fever and gastrointestinal symptoms, occurring in 80%–99.4% and 85.6%–90% of cases respectively, often presenting as abdominal pain, vomiting, or diarrhea. Severe cases may mimic acute appendicitis or peritonitis, occasionally leading to unnecessary surgeries (6, 20, 21). Additional complications include acute pancreatitis, hyperglycemia, acidosis, venous thrombosis, and electrolyte imbalances, likely resulting from immune dysregulation (22, 23) and ACE receptor distribution in the gastrointestinal tract (24); (II) Predominantly shock and left ventricular dysfunction, with incidence rates of 74% and 70%, respectively (6), which may require vasoactive agents or extracorporeal membrane oxygenation (ECMO) (25) due to myocardial damage and cytokine storms (26); (III) KD-like feature, including conjunctivitis, rash, and lymphadenopathy (17%) (6, 20), alongside potential acute kidney injury, liver damage, and neurological symptoms, though respiratory symptoms remain rare (15, 27).

**Figure 1:**
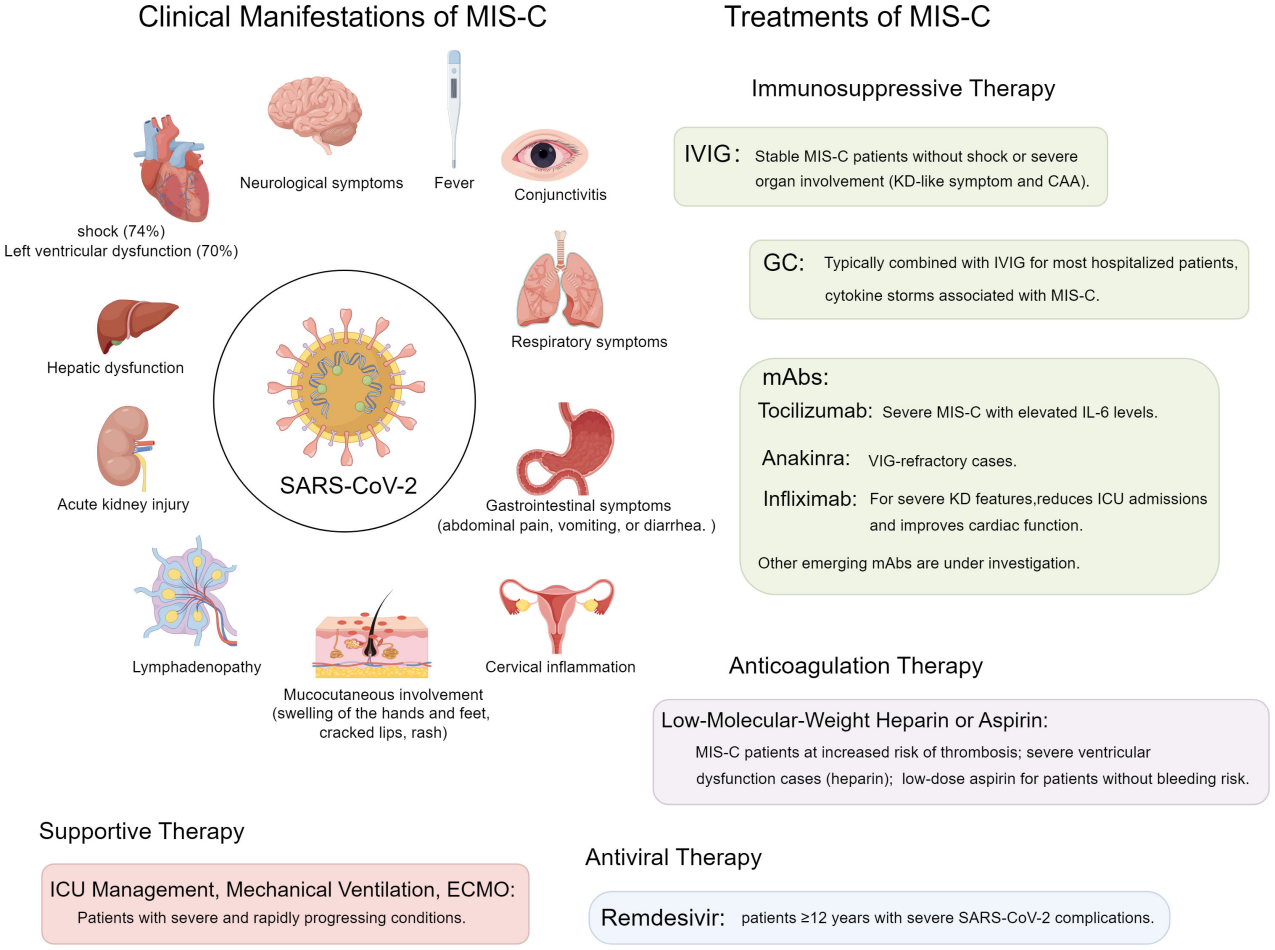
Clinical manifestations and treatment strategies for MIS-C.

Differentiating MIS-C from conditions with overlapping features, such as severe COVID-19 and KD, is crucial for appropriate management. Inflammatory factors are pivotal in inflammatory diseases progression and significantly influence the efficacy of therapies (28–31). MIS-C typically occurs in a post-infectious context, weeks after initial SARS-CoV-2 infection, and is characterized by elevated inflammatory markers like IL-6 and IL-18 (32). In contrast, severe COVID-19 presents predominantly with acute respiratory symptoms and viral pneumonia. Compared to KD, MIS-C patients are generally older, exhibit more pronounced gastrointestinal symptoms, and have a higher incidence of cardiac dysfunction and shock (33). Additionally, laboratory findings in MIS-C often show elevated markers of inflammation and cardiac injury, which can aid in distinguishing it from KD (34) (**Figure 1**).

## 4 Etiology and pathogenesis of MIS-C

The pathophysiological mechanisms of MIS-C remain inadequately understood. It may arise from direct damage induced by SARS-CoV-2, an exaggerated immune response in genetically predisposed individuals (35). SARS-CoV-2 infection can provoke endothelial damage, leading to multi-organ dysfunction (36). MIS-C onset occurs 4–5 weeks post-infection, with antibodies and viral nucleic acids absent, indicating an adaptive immune response rather than direct viral invasion (28) (15). Multiple studies (15, 37–39) demonstrate that severe immune dysregulation induces cytokine storms, causing systemic inflammation and organ failure (30–32). Alunno et al. (40) attribute MIS-C to massive inflammatory mediator release and cytokine storm activation, while Rowley et al. (39) link cytokine storms to endothelial dysfunction. The efficacy of anti-inflammatory and immunomodulatory treatments over antiviral treatment underscore excessive immune activation as the primary mechanism (40).

Although a definitive causal relationship between MIS-C and SARS-CoV-2 remains unconfirmed, the occurrence of MIS-C during COVID-19 outbreaks and its temporal correlation with SARS-CoV-2 infection suggest a strong association. The MIS-C inflammatory response resembles severe COVID-19 in adults (41–43), characterized by dysregulated cell-mediated or humoral immunity. MIS-C patients have neutralizing antibodies against SARS-CoV-2 (37), correlating with activation of IL-18, IL-16, myeloid chemotaxis, lymphocytes, monocytes, and NK cells. Autoantibodies targeting endothelial, gastrointestinal, and immune cells in MIS-C patients suggest a role in pathogenesis. In addition, SARS-CoV-2 spike protein may act as a superantigen (44), intensifying immune responses and contributing to multi-organ damage. Moreover, genetic factors may also be involved. Immunogenomic studies have identified genetic variants increasing MIS-C susceptibility, including mutations in viral recognition and antigen presentation pathways (45), and polymorphisms in immune response genes, such as those encoding cytokines and their receptors, which heighten MIS-C risk (46).

## 5 Auxiliary examinations

### 5.1 Etiological tests

All children with MIS-C test positive for serum COVID-19-specific immunoglobulin (Ig) G and IgM. In some cases, nasopharyngeal samples also test positive for COVID-19 nucleic acid (15, 47–49).

### 5.2 Laboratory examinations

In conjunction with the diagnostic criteria and clinical data for MIS-C, patients must meet at least three of the following criteria: (1) Complete blood count abnormalities, including mild to moderate anemia, decreased absolute lymphocyte count, and thrombocytosis; (2) Elevated inflammatory markers such as CRP, SF, ESR, and PCT; (3) Coagulation disturbances, including prolonged APTT/PT and increased fibrinogen; (4) Inflammatory markers; (5) Increased IL-6, IL-8, and TNF levels (47).

### 5.3 Physical examinations

Electrocardiogram findings include ST-segment depression, flattened T waves, and arrhythmias. Echocardiogram results show a reduced ejection fraction, ventricular enlargement, pericardial effusion, and coronary abnormalities; however, the severe coronary changes characteristic of KD are uncommon. Compared to KD, diminished systolic and diastolic function is observed (50). Cardiac MRI reveals hyperemia and edema without evidence of necrosis or fibrosis. Chest CT scans indicate pulmonary alterations such as decreased translucency, ground-glass opacities, or pleural effusion in approximately 50% of cases, with some patients presenting without any noticeable abnormalities (15, 49, 51). Abdominal imaging frequently detects hepatosplenomegaly, ascites or pelvic effusion, appendiceal or gallbladder enlargement, enteritis, mesenteric lymphadenitis, and thickening of the intestinal wall in 77% of cases (27). Neurological imaging often shows high-intensity T2/FLAIR signals, suggesting possible inflammatory edema (52). Echocardiography is a primary diagnostic tool for MIS-C, assessing cardiac function and coronary abnormalities with good sensitivity, though limited for subtle myocardial changes. Cardiac MRI offers higher sensitivity and specificity for inflammation and structural issues, useful when echocardiography is inconclusive. Emerging technologies like 3D echocardiography and advanced MRI enhance cardiac assessment (53).

## 6 Treatment of MIS-C

Currently, immunosuppressive therapies are broadly recommended as the primary pharmacological treatment for MIS-C worldwide. These are typically supplemented with anticoagulants such as heparin or aspirin and administered in medical facilities with ICU capabilities, mechanical ventilation, and ECMO, among other advanced therapeutic technologies (47–49, 54, 55).

### 6.1 Immunotherapy in MIS-C

The administration of intravenous immunoglobulin (IVIG) and/or glucocorticoids (GC) for treating multisystem inflammatory syndrome in MIS-C is based on the condition’s post-infectious origins, its immunological features, the elevated inflammation levels, and its clinical resemblance to KD (56). Current clinical guidelines advocate for IVIG, GC, or a combination of both as viable first-line treatments for MIS-C, but evidence remains insufficient to determine the most effective approach (57, 58). A retrospective study by Jonat et al. revealed that early administration of IVIG and GC was linked to reduced hospital stay durations, underscoring the importance of early intervention (59). Close monitoring is essential for patients receiving multiple immunomodulators, particularly those with immunodeficiencies or on immunosuppressive therapy, to balance therapeutic benefits against infection risks.

#### 6.1.1 Intravenous immunoglobulin

Given the clinical similarities between MIS-C and KD, IVIG, which is a standard treatment for KD, has also been utilized in the management of MIS-C (60). Coronary artery aneurysms (CAA) are observed in approximately 9% to 24% of MIS-C cases (47, 61, 62), and IVIG has been shown to effectively reduce CAA incidence in KD (40). A survey of MIS-C treatment practices revealed that IVIG is administered as the first-line therapy in 98% of cases (63). IVIG exerts anti-inflammatory effects by depleting neutrophils, inhibiting IL-1β, reducing complement deposition, suppressing T-cell activity, and modulating endothelial function (64, 65). Additionally, IVIG plays a crucial role in neutralizing autoantibodies, thereby facilitating their clearance from the bloodstream (66, 67).

In stable MIS-C patients without shock or severe organ involvement, IVIG monotherapy is often effective, particularly when GC are contraindicated. However, due to diagnostic challenges associated with MIS-C and the risk of delayed treatment leading to cardiogenic shock (68), IVIG is especially recommended for KD-like presentations with CAA. The typical IVIG dosing regimen involves a single infusion of 2 g/kg (with a maximum dose of 100 g), adjusted for body weight (57, 69). Repeated dosing is generally not recommended due to potential for adverse effects (57, 69). IVIG’s role in immune modulation is vital in autoimmune diseases, including MIS-C. A study from South Africa reported that most children with MIS-C received both IVIG and corticosteroids, while a smaller group required additional IVIG doses (11). For severe cases involving shock or organ failure, the combination of IVIG and corticosteroids is recommended (70, 71).

#### 6.1.2 Glucocorticoids

GC are effective immunomodulators that inhibit cytokine production through nuclear trans-repression of pro-inflammatory genes. Their non-genomic effects on both immune and endothelial cells involve modulation of membrane permeability and T-cell signaling (72, 73). GC play a crucial role in managing the cytokine storm associated with MIS-C, thereby reducing disease severity and mitigating tissue damage (74, 75). Early GC administration within 48 hours of hospitalization has been associated with shorter stays, suggesting that prompt initiation is critical (59).

The standard GC regimen typically consists of short-term treatment with methylprednisolone at a dose of 1–2 mg/kg/day for 3–5 days (76, 77). In some cases, high-dose protocols, such as 30 mg/kg/day for three days, can be tapered over a period of two weeks (76). Licciardi et al. reported a 67.7% response rate in children treated exclusively with intravenous methylprednisolone (78). GC combined with IVIG is standard for most hospitalized MIS-C patients unless there are contraindications to GC use (79), with tapering over 2–3 weeks or longer (57). In severe cardiovascular involvement, combined IVIG and GC therapy may resolve complications rapidly (14). For cases resistant to standard treatments, GC pulse therapy or biologic agents may be necessary (57).

#### 6.1.3 Specific monoclonal antibodies

The pathophysiology of MIS-C involves elevated pro-inflammatory cytokines, particularly IL-6 (80–83). Coronaviruses upregulate IL-6 via enhanced mRNA transcription and stabilization (84, 85), contributing to cytokine storm syndromes (86) and adverse outcomes like prolonged viral shedding and respiratory failure (87–89). Tocilizumab, an IL-6 inhibitor, is the preferred therapy, administered initially at 4–8 mg/kg or 400 mg in 100 ml saline intravenously over >1 hour, with a possible second dose after 2 hours, not exceeding 800 mg or two doses total (90). This treatment demonstrates substantial effectiveness and safety (84, 85, 91). Severe MIS-C often requires combined IVIG, GC, and tocilizumab (15, 92).

Anakinra, an IL-1 inhibitor, was initially used due to MIS-C’s association with KD but is now reserved for IVIG-refractory cases (15, 91). SARS-CoV infection induces IL-1 production by alveolar type II cells (85, 93), and MIS-C patients exhibit increased IL-1β gene expression (94). Anakinra can block IL-1 (95), benefiting patients with severe sepsis and macrophage activation syndrome (96, 97), serving as an alternative therapy for IVIG-refractory MIS-C (57, 98). It is typically administered at 2–3 mg/kg subcutaneously (69) or intravenously if necessary (97). If no response within 24–48 hours, switching to tocilizumab or infliximab is recommended (69). TNF-α is implicated in severe KD (99, 100), and infliximab, a TNF-α mAb (5–10 mg/kg IV) (47, 101, 102) reduces ICU admissions, enhances cardiac function (58), and facilitates corticosteroid tapering (57, 103). Eculizumab, an anti-C5 mAb, inhibits terminal complement activation (104), improving MIS-C-related thrombotic microangiopathy (105). Emapalumab blocks IFN-γ and tadekinig alfa inhibits IL-18 (106), but current data on these agents remain limited. Emerging therapies, including new mAbs targeting various cytokines and immune pathways such as baricitinib and JAK inhibitors, are under investigation to mitigate cytokine signaling and reduce inflammation in MIS-C patients (107, 108).

### 6.2 Anticoagulation therapy and antiviral therapy

The hypercoagulable state in MIS-C requires careful anticoagulation management (109, 110). Early heparin therapy is essential for children with severe ventricular dysfunction, and low-molecular-weight heparin or aspirin is recommended for those at high risk of thrombosis (110, 111). For MIS-C patients without bleeding risks, low-dose aspirin is advised unless contraindicated, continuing for at least one month post-diagnosis or until inflammatory markers normalize (35, 57, 112–115). Antiviral agents such as ribavirin, interferon, and lopinavir/ritonavir have uncertain efficacy for MIS-C, as it is immune-mediated rather than viral cytopathic (77, 116). Remdesivir is used for severe SARS-CoV-2 cases in patients ≥12 years but has unclear relevance in MIS-C; consultation with specialists is recommended for refractory cases (117). Chloroquine phosphate’s efficacy in MIS-C is unproven (47). Due to the severity of MIS-C, ICU admission for respiratory, cardiac, and circulatory support is often necessary, with ECMO in critical cases (15, 47–49) (**Figure 1**).

## 7 Prognosis of MIS-C

Despite severe presentations, the MIS-C mortality is low (1.9%), and most patients recover with timely treatment (20, 118). Coronary artery abnormalities (9%-24%) warrant regular follow-up (2, 119). Additionally, MIS-C may lead to long-term neurodevelopmental impacts, including cognitive and behavioral changes (120), and chronic inflammatory conditions that cause sustained immune dysregulation, increasing risks for autoimmune disorders and prolonged systemic inflammation (121). Consequently, long-term monitoring and management are essential for comprehensive patient care. Furthermore, IL-18 and TNF-α serve as prognostic markers for disease severity and the efficacy of IVIG therapy in MIS-C (122). Endothelial-cell-specific molecule-1 is a valuable diagnostic and prognostic biomarker, aiding in identifying MIS-C patients at higher cardiovascular risk and guiding treatment strategies (123).

## 8 Conclusion

In conclusion, MIS-C is a severe complication of SARS-CoV-2 in children, marked by systemic inflammation and multi-organ involvement. Its pathogenesis involves immune dysregulation, with delayed antibody and cytokine responses. Early recognition and a multidisciplinary approach, including IVIG, corticosteroids, and biologics, are critical for effective management. Prognosis is generally favorable with timely treatment, though long-term monitoring for cardiovascular sequelae is essential. Future research should focus on understanding its immunopathology, refining diagnostics, and optimizing therapies to improve outcomes.
